# Beyond the Brake: the Subthalamic Nucleus Predominantly Facilitates Action in Non-human Primates

**DOI:** 10.64898/2025.12.09.692702

**Published:** 2025-12-12

**Authors:** Atsushi Yoshida, Richard J. Krauzlis, Okihide Hikosaka

**Affiliations:** 1 Laboratory of Sensorimotor Research, National Eye Institute, National Institutes of Health, Bethesda, MD 20892, USA; 2 Integrated URA Office, Hokkaido University, Sapporo, 001-0021, Hokkaido, Japan; 3 Lead Contact

**Keywords:** Subthalamic nucleus (STN), Basal ganglia, Action initiation, Motor control, Saccade generation

## Abstract

The subthalamic nucleus (STN) is traditionally viewed as a brake on motor output. Challenging this model, we investigated its role in value-based decision-making by recording single-neuron activity from the primate STN during a choice task. Our findings reveal that the STN contains functionally distinct clusters of neurons and, contrary to its classical role, the vast majority of task-related neurons (approximately 89%) exhibited activity consistent with action facilitation. Two of the clusters contained facilitative neurons that increased their firing for rewarded choices, and critically, their response latency predicted saccadic reaction times on a trial-by-trial basis. Population-level analyses further demonstrated that these facilitative neurons robustly encode the upcoming choice. A smaller third cluster of neurons showed activity consistent with action suppression. These results indicate a dual function of the STN, suggesting it plays a predominant role in actively facilitating desired actions, rather than simply suppressing unwanted ones.

## Introduction

The subthalamic nucleus (STN) is a crucial component of the basal ganglia circuitry that has traditionally been viewed as responsible for suppressing motor output, a concept rooted in classical models of the indirect and hyperdirect pathways.^1–10^ This “brake” model is supported by extensive anatomical, physiological, and lesion studies. However, this classical view is increasingly challenged by emerging evidence suggesting a more complex and multifaceted role. Studies across species now implicate the STN in aspects of behavioral control beyond inhibiting actions. For instance, optogenetic studies in rodents demonstrate that STN stimulation can elicit rapid movements,^11,12^ while primate neurophysiology shows STN activity is modulated during action switching and value-based decisions.^13–15^ Furthermore, human neuroimaging reveals that STN activity dynamically adapts to behavioral context,^16,17^ and clinical findings from deep brain stimulation (DBS) in Parkinson’s disease show complex effects on both motor control and impulsivity.^18–20^ Taken together, these findings suggest the STN’s role extends far beyond simple motor suppression.

A parallel debate surrounds the STN’s principal target, the globus pallidus external segment (GPe). Also traditionally viewed as an inhibitory station, a growing body of evidence now implicates the GPe in action facilitation.^21–24^ In a previous study using the same behavioral paradigm as the present work, we identified a distinct GPe neuronal population that facilitates action initiation and demonstrated that this function causally depends on excitatory inputs.^25^ Given the dense excitatory projection from the STN to the GPe,^26,27^ these findings led us to hypothesize that the STN provides the crucial excitatory drive underlying this facilitative function in the GPe, a direct contradiction to its canonical role.

The primary objective of this study was to test a fundamental prediction of this hypothesis: that the STN itself contains a neuronal population whose activity is consistent with a role in action facilitation. To this end, we recorded single-neuron activity from the STN in monkeys performing a value-based choice task, allowing for a direct comparison with our previous GPe findings. Our results reveal that the STN contains a substantial population of neurons with facilitative rather than inhibitory properties. More broadly, these results contradict the traditional model of basal ganglia function and instead support an updated framework that recasts the STN not just as a brake, but as a dynamic controller of voluntary action.

## Results

### Behavioral performance

**Figure 1.**
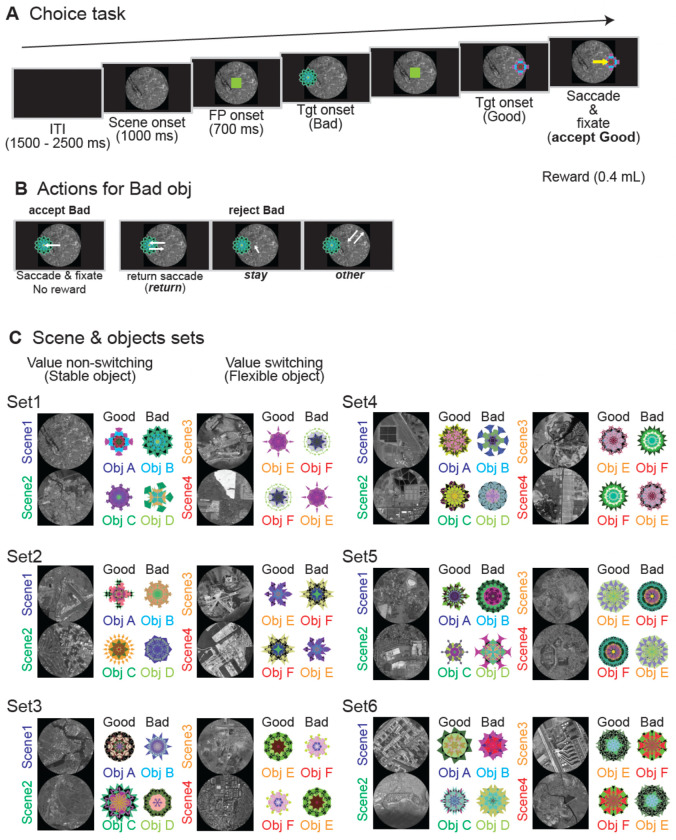
The sequential choice task. (A) Trial sequence. Each trial began with the presentation of a background scene (1000 ms) and a central fixation point (FP, 700 ms). A single object, either “good” (rewarded) or “bad” (non-rewarded), was then presented at one of six peripheral locations. Monkeys received a liquid reward for making a saccade to and fixating on a good object. (B) Response options for bad objects. When a bad object was presented, monkeys could reject it using one of three strategies: a “return” saccade (looking at the object only briefly before making a saccade back to the center), “stay” (maintaining central fixation), or “other” (looking away from both the object and center). Incorrectly accepting a bad object resulted in no reward. (C) Context-dependent value assignment. Six different sets of objects and scenes were used across sessions. Within each set, Scenes 1 and 2 featured objects with stable values. In Scenes 3 and 4, the same pair of objects was used, but their reward contingencies were reversed (“flexible value”), requiring the monkeys to adapt their choices based on the scene context.

The two monkeys (C and S) performed a sequential choice task (Figure 1A) in which they decided whether to accept a “good” (rewarding) object or reject a “bad” (non-rewarding) object. To reject a bad object, they could employ one of three strategies when a bad object was presented: they could generate a “return” saccade back to fixation after briefly glancing at the bad object, they could “stay” at the central fixation point without looking, or they could look at “other” locations in the visual scene (Figure 1B). During each recording session, one of six different sets of objects and scenes was randomly selected (Figure 1C). To dissociate neural activity related to learned value from responses to simple visual features, the design included objects with stable values as well as a “flexible value” condition. In this flexible condition (Scenes 3 and 4), the reward contingencies for the same pair of objects were reversed, requiring the monkeys to adapt their choices based on the scene context.

Consistent with our previous studies using this paradigm,^25,28,29^ the monkeys’ performance demonstrated a stable and robust understanding of the task rules and object values. Saccadic reaction times (RTs) were substantially faster for accepting good objects compared to rejecting bad objects via a return saccade (Figure S1A; Welch t-test, *P* < 0.0001). When faced with a bad object, the monkeys predominantly chose the “return” strategy over the “stay” strategy (Figure S1B). This behavioral pattern, which provides a reliable basis for analyzing neuronal activity related to action selection, was consistent across all recording sessions, and the same as in our previously published work.^25,28,29^

### General Firing Properties of STN Neurons

We recorded the activity of 187 task-responsive single neurons in the STN of two monkeys (Monkey C: 90; Monkey S: 97) as they performed the choice task. The subsequent analyses focused on trials with contralateral object presentations, which consistently elicited the most robust neuronal responses.

**Figure 2.**
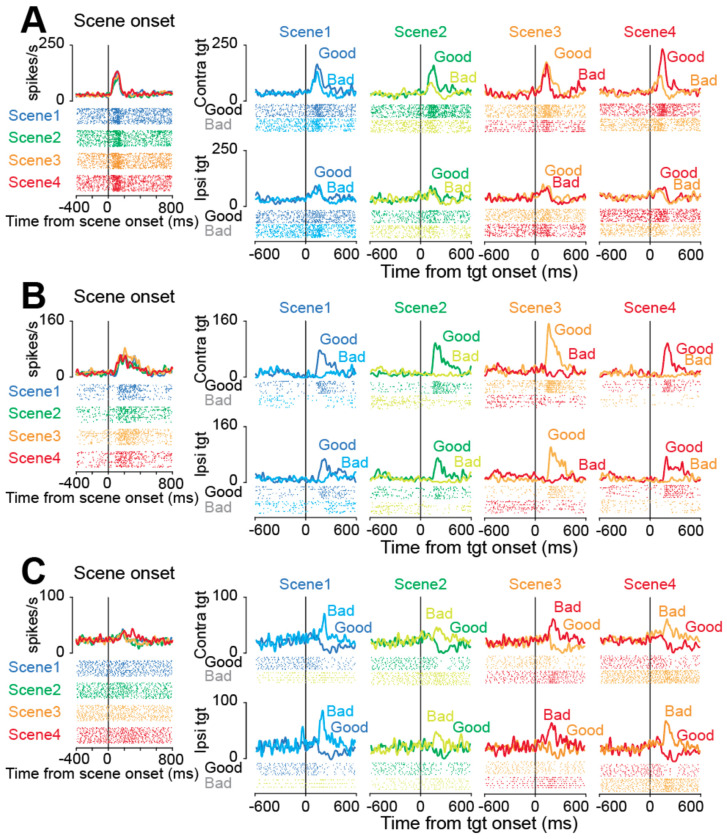
Representative neurons of the three clusters. (A, B, C) The raster plots and histograms of representative three neurons. Data are aligned with the scene onset or target onset (vertical line in each panel). Traces in different colors indicate the spike density for scene onset or for good or bad objects in each scene.

We observed a variety of firing patterns across our population of STN neurons in the sequential choice task. Figure 2 illustrates this variety with three representative single neurons. The most prominent and consistent responses across the population were sharp, phasic changes in firing rate immediately following target onset. However, the stimulus preferences in these responses varied across neurons. For example, some neurons increased their activity for both good and bad objects, with a preference for good objects (Figure 2A). Other neurons responded strongly to good objects but with little or no modulation for bad objects (Figure 2B). Finally, some neurons exhibited the opposite preferences and responded strongly to bad objects and were inhibited by good objects (Figure 2C).

This observed heterogeneity in firing patterns indicates that the STN is not a functionally uniform structure but instead contains distinct neuronal subpopulations, each potentially contributing differently to the decision-making process. To examine this possibility and objectively classify the neurons based on their activity profiles, we proceeded with a clustering analysis.

### Classification of STN Neurons into Functional Clusters

To objectively classify the diverse activity profiles observed in the STN, we used a k-means clustering algorithm based on the standardized firing rates during the presentation of contralateral good and bad objects (100–300 ms post-object onset). This specific methodology and time window were chosen to be identical to those used in our preceding study of the GPe, thereby allowing for a direct comparison with the functionally analogous neural populations we identified in the GPe.^25^ To quantitatively determine the optimal number of clusters (K), we simulated the silhouette values 5,000 times and found that K=3 produced the largest average silhouette value (Figure S2A). Accordingly, this data-driven analysis robustly partitioned the neuronal population into three distinct functional clusters (Figure S2B): Cluster 1 (n = 76), Cluster 2 (n = 90), and Cluster 3 (n = 21). We then examined whether these functionally defined groups also possessed distinct physiological or anatomical characteristics. Unlike our previous findings in the GPe, we found no significant difference in the baseline firing rates among the three STN clusters (Figure S2C; Kruskal-Wallis test, *P* > 0.05). Furthermore, 3D reconstruction confirmed that our recordings were primarily located in the ventral STN (Figure S2D).

### Task-Related Activity Revealed Distinct Value and Spatial Coding

**Figure 3.**
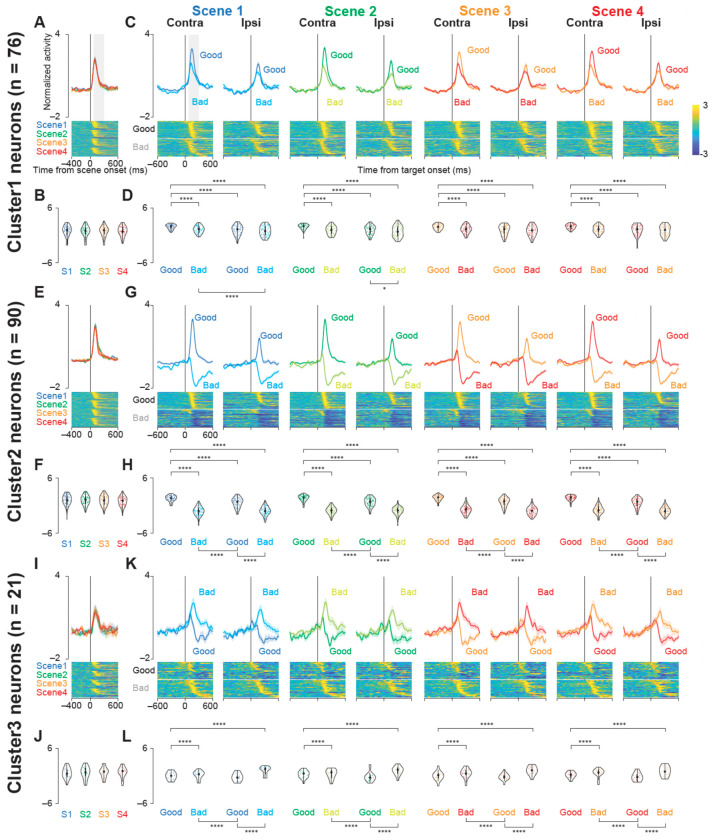
Population activity of three groups of STN neurons at scene and target onsets during the choice task. (A, E, I) Mean normalized population activity (activity was normalized by subtracting the baseline firing rate and dividing it by the standard deviation) of Cluster 1 (A), Cluster 2 (E), and Cluster 3 (I) neurons aligned to the onset of the scene (scenes 1–4) during the choice task. The shaded areas indicate mean ± standard errors of mean (SEMs). The lower panels show color maps of the normalized activity of individual neurons. Each row in the color map represents a single neuron, and the neurons are sorted based on the time at which their standardized activity exceeds a threshold. (B, F, J) Violin plots showing the distribution of mean normalized neuronal activity of individual neurons in Clusters 1 (B), 2 (F), and 3 (J) for each scene onset during the choice task. Neuronal activity was measured at 200-ms intervals beginning 100 ms after scene onset. The larger circle indicates the median value, the thick vertical line shows the interquartile range (IQR), and the thin vertical line indicates 1.5 × IQR. (C, G, K) Mean normalized population activity of Cluster 1 (C), Cluster 2 (G), and Cluster 3 (K) neurons aligned to the onset of contralateral and ipsilateral good and bad targets for scenes 1-4. (D, H, L) Violin plots showing the distribution of the mean normalized neuronal activity of individual neurons in Clusters 1 (D), 2 (H), and 3 (L) for each target-onset condition. Neuronal activity was measured for a 200-ms interval beginning 100 ms after target onset. The asterisks indicate significant differences in neuronal activity among the four conditions (contra good, contra bad, ipsi good, ipsi bad) within each scene (post hoc pairwise t tests with Bonferroni correction, *P* < 0.05, etc.). See also Tables S3–S5.

To dissect the functional roles of these clusters, we examined their population activity aligned to two critical task events: scene onset and object onset (Figure 3). As would be expected from our clustering method, the activity of the neurons in each cluster was clearly different following the presentation of the target objects. In contrast, none of the clusters showed significant modulation related to the scene context itself (Figure 3A, B, E, F, I, J). We therefore characterized the specific nature of this target-evoked activity for each of the three clusters.

The activity of Cluster 1 neurons increased following the presentation of both good and bad objects, though the response was significantly stronger for good objects (Figure 3C, D; *P* < 0.001, see Table S3 for statistics). These neurons also showed a clear preference for contralateral targets, indicating that they integrated both value and spatial information.

Cluster 2 exhibited a distinct, bidirectional response profile. Upon target presentation, these neurons increased their firing for good objects but decreased their firing for bad objects (Figure 3G, H; *P* < 0.001, Table S4). This opponent coding of positive and negative value, with a strong response to contralateral good targets, points to a primary role for this cluster in value-based action selection.

In contrast, Cluster 3 appeared to preferentially encode negative outcomes. While their activity increased for both object types, the response was significantly more pronounced for bad objects compared to good objects (Figure 3K, L; *P* < 0.001, Table S5). This response pattern is consistent with a role in signaling the need to suppress or reject an action.

Together, these findings demonstrate that the STN contains functionally specialized subpopulations, each processing a unique combination of value and spatial information to guide behavior.

### Validation of Functional Groupings with a Data-Driven PCA Approach

To validate the functional groupings identified by our window-based method and to explore the underlying structure of the data in a more objective, data-driven manner, we next employed Principal Component Analysis (PCA). To focus on the evoked responses that are central to this study, we performed this analysis on the standardized neural activity from a short window of 0 to 300 ms after the onset of contralateral good and bad objects.

This analysis revealed the principal temporal patterns (components) that explain the variance across the neural population (Figure S3A), with a Scree plot indicating that most of the variance was captured by the first few components (Figure S3B). We then performed k-means clustering on the weights of each neuron on the top three PCs (PC1-3), which can be visualized by considering the position of each neuron in a 3-dimensional functional space (Figure S3C).

To fully characterize the possible functional groupings, we examined the solutions for both K=2 and K=3. The results of this analysis are shown in Figure S4, which displays the population activity and the normalized neuronal activity of individual neurons.

Both clustering solutions consistently support our primary findings. The K=2 solution revealed a fundamental division of the neurons into a ‘value-coding facilitative’ group and a larger ‘general facilitative’ group. When the number of clusters was increased to K=3, the analysis largely preserved the distinct ‘value-coding’ group, while subdividing the ‘general facilitative’ group into two smaller sub-groups.

Taken together, this data-driven analysis is consistent with our main conclusion that the activity of STN neurons in our task is organized around principles of action facilitation. The core distinction between value-coding and general facilitation was observed regardless of the clustering details, lending further support to our claim that the STN’s role in this context is largely facilitative. Notably, a functionally distinct suppressive group, analogous to Cluster 3 from our initial analysis, was not robustly isolated by this method, a point we will consider further in the Discussion.

### Saccade-Related Activity Reflected Action Choice

**Figure 4.**
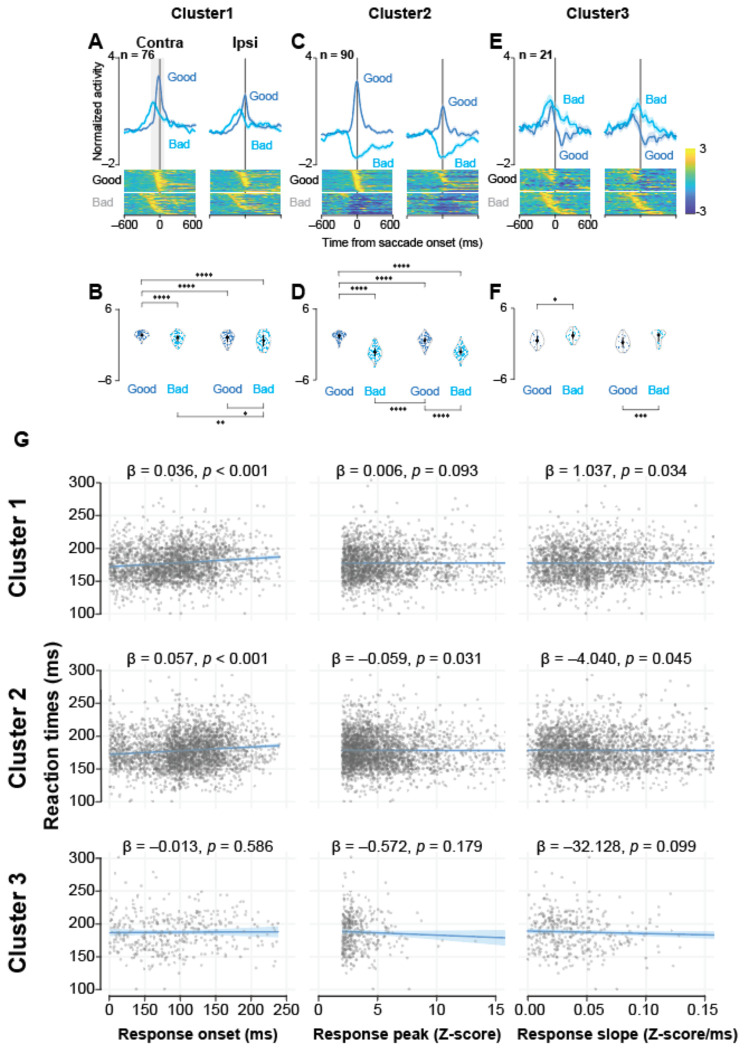
The population activity of the three groups of STN neurons aligned with saccade onset during the choice task. Note that because the reaction times for rejecting ”bad” objects were longer than those for accepting “good” objects (see Figure S1A), stimulus-related activity for “bad” trials appears shifted earlier in time relative to saccade onset. This figure illustrates activity relative to the motor event, not the stimulus-evoked response latency. (A, C, E) Mean normalized population activity of Cluster 1 (A), Cluster 2 (C), and Cluster 3 (E) neurons aligned to saccade onset for contralateral or ipsilateral, and good or bad objects in Scene 1 during the choice task. The shaded areas indicate ± SEMs. The lower panels show color maps of normalized neuronal activity of individual neurons, with each row representing a single neuron, sorted as in Figure 2. (B, D, F) Violin plots showing the distribution of the mean normalized neuronal activity of individual neurons in Clusters 1 (B), 2 (D), and 3 (F) when monkeys made a saccade to the target. Neuronal activity was measured at 200-ms intervals from 150 ms before to 50 ms after saccade onset. The asterisks indicate significant differences in neuronal activity among the four conditions (*P* < 0.05, etc.; post hoc pairwise t tests with Bonferroni correction). See also Tables S6–S8. (G) Trial-by-trial relationship between STN neuronal activity and reaction times. Scatter plots showing the relationship between saccadic RTs and three neuronal response parameters for each STN cluster. Each gray dot represents a single trial under the ‘good, contra, accept’ condition. Rows and columns correspond to the three neuronal clusters and three response parameters (onset latency, peak magnitude, and slope), respectively. Lines and shaded ribbons represent the predicted marginal effect and 95% confidence interval, respectively, derived from the LMM. Inset values indicate the unstandardized regression coefficient (β) and the corresponding *P*-value from the LMM. *P*-values shown are uncorrected; interpretation in the main text is based on a Bonferroni-corrected significance threshold (*P* < 0.0056). Full model results are provided in Table S9.

To simplify the analysis of movement-related activity, we focused on data from Scene 1, as target-aligned responses were consistent across all scenes. To investigate the relationship between neuronal activity and movement execution, we next aligned the firing of each cluster to saccade onset (Figure 4).

The activity of neurons in Cluster 1 was significantly elevated around the time of saccade initiation, with the highest firing rates observed for saccades to contralateral good objects (Figure 4A, B; *P* < 0.001, see Table S6 for statistics). This robust peri-movement activity suggests a direct contribution to the generation of saccades.

Neurons in cluster 2 also showed strong modulation at saccade onset, but their activity was again contingent on value. Firing increased for saccades to good objects and decreased for those to bad objects (Figure 4C, D; *P* < 0.001, Table S7), reinforcing the idea that neurons in this cluster integrate value signals directly into motor execution commands.

In contrast, neurons in cluster 3 showed the greatest increase in activity preceding saccades to bad objects (Figure 4E, F; *P* < 0.001, Table S8). This response pattern, which is opposite to that of the facilitative clusters, is consistent with a role in signaling the need to override or suppress an unwanted movement.

### Activity in Facilitative Clusters Was Associated with Reaction Time

The preceding analyses suggest that neurons in clusters 1 and 2 are involved in facilitating saccades. If this is the case, then trial-to-trial fluctuations in their neuronal activity should be associated with corresponding variations in reaction time (RT). To examine this, we analyzed the relationship between RT and three neuronal response parameters (onset latency, peak magnitude, and slope) using LMMs (see Table S9 for full model results). This approach allowed us to account for non-independent observations from the same monkeys and neurons. To correct for multiple comparisons across the nine models fitted, we applied a Bonferroni correction, adopting a significance threshold of *P* < 0.0056 (0.05/9).

After applying this correction, the LMM analysis indicated that only the response onset latency in the facilitative clusters had a statistically significant, albeit modest, association with RT. For both Cluster 1 (β = 0.036, *P* = 0.0004) and Cluster 2 (β = 0.057, *P* < 0.0001), a later response onset was significantly associated with a longer RT.

In contrast, neither the peak firing magnitude nor the response slope for these facilitative clusters showed a significant relationship with RT after Bonferroni correction (all *P* > 0.0056). Furthermore, consistent with the uncorrected analysis, no activity parameters from Cluster 3 significantly predicted RT.

Taken together, these results suggest a subtle link between the trial-by-trial activity in facilitative, but not suppressive, STN populations and the speed of action initiation. Notably, after rigorous statistical correction, this weak association appears to be specific to the timing of the neuronal response, as only the onset latency, and not the response magnitude or slope, remained a significant predictor of behavior.

### Single-Neuron and Population-Level Encoding of Choice

**Figure 5.**
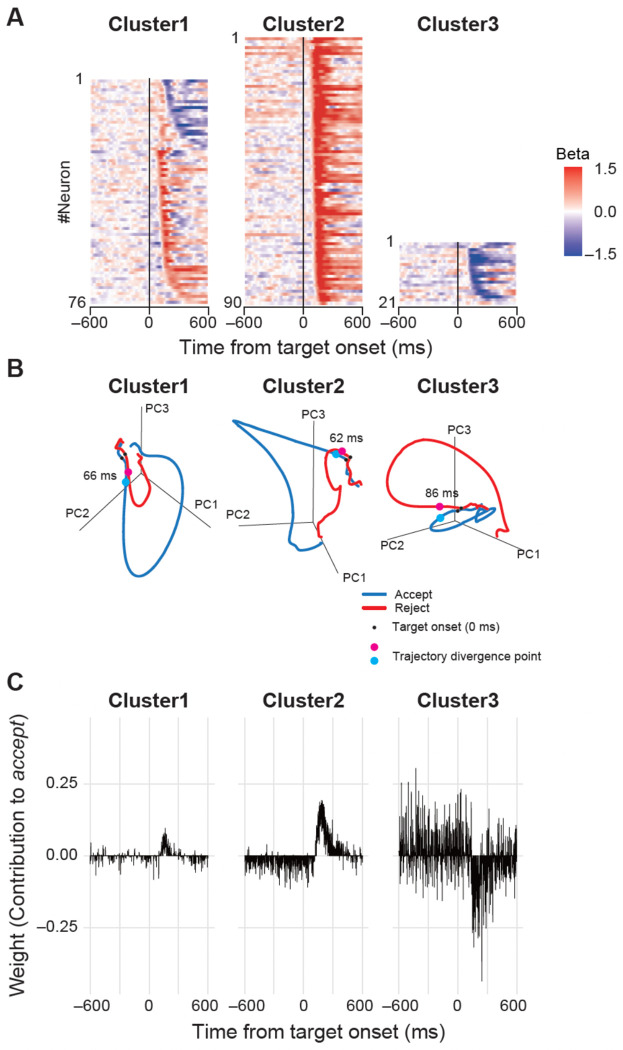
Single-neuron and population-level encoding of choice. (A) Single-neuron choice encoding. Heatmaps show the time course of β coefficients from a sliding-window GLM for each neuron. Red indicates a positive β (encoding ‘accept’), while blue indicates a negative β (encoding ‘reject’). Neurons within each cluster are sorted by their response preference and latency. (B) Population activity trajectories. The evolution of the population activity for each cluster is visualized as a trajectory in a 3D state space derived from PCA. Traces for ‘accept’ (blue) and ‘reject’ (red) choices are shown. The black dot indicates target onset (t = 0), and the cyan and magenta dots indicate the time at which the two trajectories became statistically separable. (C) Population decoding weights for each cluster. The time course of weights from three separate Elastic Net classifiers, each trained to predict choice using the population activity from neurons within Cluster 1, 2, and 3, respectively. A positive weight indicates that neuronal activity at a given time point contributes to predicting an ‘accept’ choice, whereas a negative weight contributes to predicting a ‘reject’ choice.

To quantify how strongly individual STN neurons encoded the monkey’s choice over time, we performed a trial-by-trial analysis using a sliding-window general linear model (GLM) for contralateral trials (Figure 5A). In this analysis, the mean neuronal activity within a 50-ms window was regressed against the upcoming choice (‘accept’ = 1, ‘reject’ = −1). The resulting heatmaps of β coefficients in Figure 4A revealed distinct patterns across clusters. A large majority of neurons in Cluster 1, and nearly all in Cluster 2, showed positive β coefficients, robustly encoding the ‘accept’ choice. In contrast, most neurons in Cluster 3 exhibited negative β coefficients, consistent with encoding the ‘reject’ choice.

To understand how these individual signals combined and evolved collectively, we next visualized the population activity using PCA on the trial-averaged spike density functions. This approach visualizes the population activity as neuronal trajectories in a low-dimensional state space (Figure 5B).

In all clusters, the trajectories for ‘accept’ and ‘reject’ choices began to separate into distinct paths shortly after the target appeared. We quantified this separation and found that the neuronal representations diverged remarkably early (62–86 ms post-target onset), well before the average saccadic reaction times. This indicates that the STN population activity reflects the outcome of the decision-making process itself, rather than simply encoding the subsequent motor command.

To quantify the predictive power of the STN population, we performed a decoding analysis using an Elastic Net classifier (Figure 5C). To obtain an unbiased performance estimate and prevent data leakage from non-independent trials, we employed a nested, 10-fold cross-validation scheme where folds were grouped by neuron (see Methods). When analyzing the pooled data from both monkeys, the model predicted the action choice with an accuracy of *AUC* = 0.747 and, to a lesser extent, also predicted RTs (cross-validated *R^2^* = 0.062).

To assess the relative importance of each cluster, we performed both ablation and single-cluster analyses. The results revealed a critical and dominant role for Cluster 2. In the ablation analysis, removing Cluster 2 from the model caused performance to drop precipitously to an *AUC* of 0.558, a level barely above chance. Conversely, removing either Cluster 1 (*AUC* = 0.807) or Cluster 3 (*AUC* = 0.806) slightly improved the overall performance. This conclusion was further strengthened by the single-cluster analysis. A model using only Cluster 2 neurons achieved a remarkably high accuracy of *AUC* = 0.918, while models using only Cluster 1 (*AUC* = 0.614) or Cluster 3 (*AUC* = 0.607) neurons performed substantially worse.

Crucially, these key findings were highly consistent when the analysis was repeated for each monkey individually. For both Monkey C and Monkey S, a model using only Cluster 2 neurons achieved high accuracy (*AUC* = 0.885 and 0.916, respectively), while removing Cluster 2 consistently caused a dramatic drop in performance (*AUC* = 0.519 and 0.573, respectively).

Taken together, these population-level analyses provide strong quantitative support for a functional division of labor within the STN. The predictive information for value-based choice is not distributed evenly across populations but is instead overwhelmingly concentrated in the Cluster 2 neurons, which powerfully signal the facilitation of a desired action.

## Discussion

In the present study, we investigated the role of the primate STN in value-based decision-making. Our findings reveal that the STN is not a functionally homogeneous structure but is composed of distinct functional groupings. Critically, the vast majority of task-related neurons exhibited activity consistent with action facilitation, challenging the traditional view of the STN as primarily a brake on motor output. These results suggest the STN plays a multifaceted role, with a predominant function in the facilitation of desired actions, while also containing a component related to the suppression of unwanted ones.

### A Dual Role for the STN in Action Control

A central finding of this study is the prominent role of the STN in action facilitation, a function that contradicts its classical depiction as a purely suppressive structure. The two facilitative populations (Clusters 1 and 2) constituted the majority of task-related neurons (approximately 89%), a finding consistent with other primate studies reporting a large proportion of STN neurons that increase their activity during movement.^30,31^ While the dual role of STN neurons in movement execution and cancellation has been shown,^14^ our task required a higher-order decision process involving the selection of a high-value action from multiple options. The fact that facilitative neurons constituted an overwhelming majority in our data underscores that the STN’s role extends beyond simple Go/Stop signaling, highlighting a crucial function specifically in the selection and active facilitation of value-based actions.

The most compelling evidence for this facilitative role comes from our trial-by-trial analysis, which revealed a direct correlation between the onset latency of neuronal activity in these clusters and saccadic reaction times. This relationship suggests that the STN contributes not only to what action is selected but also to when it is initiated. Furthermore, our population-level analysis showed that the neuronal trajectories for ‘accept’ and ‘reject’ choices diverged remarkably early, well before movement onset, indicating that the STN is involved in the decision-making process itself, rather than simply relaying a pre-determined motor command. This facilitative function appears to be a conserved, cross-species principle. Indeed, our findings are remarkably consistent with ‘type 1‘ neurons identified in mice, whose activity was essential for sustaining locomotion.^12^ Our result, specifically that the response latency of our facilitative clusters predicts saccadic reaction times, suggests that this facilitative function generalizes to value-based decision-making in primates.

While our window-based analysis revealed a predominant facilitative function, it also identified a smaller group of neurons (Cluster 3) whose activity was consistent with the classical role of the STN in action suppression.^5,6,7^ To further validate this functional architecture with a more objective, data-driven approach, we also applied PCA. This analysis revealed a consistent organizational principle: a primary division of the STN population into two major facilitative profiles, one strongly encoding value and another that provided a more general facilitative signal. Notably, a purely suppressive group analogous to the one identified by our initial method was not isolated. Although one of the K=3 groupings did exhibit a slight preference for ‘bad’ objects at the population level, this group was functionally mixed and lacked the distinct suppressive profile of the cluster identified in our initial analysis. This suggests that while a suppressive component is present in STN activity, it may not define a separable class of neurons, further underscoring that the STN’s role is predominantly facilitative. It is also possible that the suppressive activity observed in Cluster 3 reflects more than a simple motor brake. This activity could represent a higher-order cognitive signal related to conflict monitoring or the signaling of unexpected outcomes (i.e., the appearance of a ‘bad’ object), consistent with the broader role of the STN in cognitive control. Therefore, our results suggest the classical model can be placed within a broader framework. In this view, the STN operates as a dynamic controller, with a function that appears to be largely facilitative, acting as an ‘accelerator’ for desired actions.^35^

### The Potential STN-GPe Facilitative Pathway and its Clinical Implications

The functional architecture we observed with our initial window-based analysis shows a notable parallel to the subpopulations we previously identified in the GPe using the identical paradigm.^25^ This correspondence strengthens the hypothesis that the STN provides a major source of excitatory, facilitative drive to the GPe. This notion is supported by several lines of evidence. First, our recordings were concentrated in the ventral STN and dorsal GPe, respectively, two regions known to be tightly interconnected in the primate.^26^ Second, the response properties of the facilitative STN clusters are analogous to facilitative GPe populations, which in turn resemble prototypical GPe neurons. This aligns with findings in rodents showing that STN projections preferentially target these prototypical neurons implicated in movement promotion.^24^

However, it is important to acknowledge that the STN is not the only candidate for this role. For instance, a direct corticopallidal pathway is another significant source of excitatory input,^36–38^ and our current findings do not exclude its potential contribution to the facilitative GPe activity.

The recognition of this substantial STN-GPe facilitative pathway has significant clinical implications. For example, the complex effects of STN deep brain stimulation (DBS) in Parkinson’s disease, which can improve motor facilitation as well as inhibition,^39–41^ are difficult to explain using conventional models alone. Our findings suggest that the therapeutic effects of DBS may be mediated not only via the suppressive STN-SNr pathway but also through the facilitative STN-GPe pathway we describe. Therefore, incorporating this circuit into existing theory is crucial for a more complete understanding of both normal basal ganglia function and the pathophysiology of related movement disorders.

### Limitations and Future Directions

The present study has several limitations. First, our findings are correlational; while we demonstrate a strong link between STN activity and behavior, we have not established a causal relationship. Second, the functional analogy between STN and GPe populations does not constitute direct proof of a monosynaptic circuit. It remains possible that other excitatory inputs, for instance from the cortex, also contribute to the facilitative responses observed in the GPe.

Future studies employing circuit-dissection tools are essential to address these limitations. Establishing causality will require techniques such as optogenetics or chemogenetics (e.g., DREADDs) to selectively manipulate the activity of specific STN clusters (e.g., the facilitative populations) while observing the impact on both GPe neuronal activity and behavior. Furthermore, pathway-specific viral tracing methods will be necessary to anatomically map the projections from each functional STN cluster to their precise targets within the GPe and other basal ganglia nuclei. Such experiments will be crucial not only for validating the proposed STN-GPe facilitative circuit but also for delineating its functional role relative to the canonical direct pathway, which also contributes to action promotion. Ultimately, integrating these novel pathways into established models of the basal ganglia is an important and necessary step toward a more complete understanding of how the brain controls voluntary movement.

## Resource availability

### Lead Contact

Further information and requests for resources and reagents should be directed to and will be fulfilled by the lead contact, Atsushi Yoshida (yoshidaatsushi0113@gmail.com).

## Methods

### EXPERIMENTAL MODEL AND STUDY PARTICIPANT DETAILS

#### Subjects

All experimental procedures were approved by the National Eye Institute Animal Care and Use Committee and conducted in accordance with the Public Health Service Policy on Laboratory Animal Care. Two male rhesus macaques (Macaca mulatta, 8-10 kg), designated as Monkeys C and S, served as subjects. These animals were also used in our previous studies.^25,28,29^ Surgical procedures were performed under isoflurane anesthesia and aseptic conditions to implant a plastic head holder and recording chambers. After a recovery period, the monkeys were trained to perform oculomotor tasks. During experimental sessions, the monkeys’ heads were held in a fixed position, and we monitored their eye movements at 1000 Hz using an infrared eye-tracking system (EyeLink 1000, SR Research). To maintain motivation, fluid intake was regulated throughout the experimental period. Detailed descriptions of the surgical and postoperative care have been published previously.^28^

### METHOD DETAILS

#### Behavioral Task and Visual Stimuli

Experiments took place in a light- and sound-attenuated room, with visual stimuli presented on a screen via an LCD projector (PJ658, ViewSonic). Task control and data acquisition were managed by custom C++ software. The choice task paradigm was identical to that used in our previous work.^28^

In each session, one of six scene-object sets was chosen at random. Each set contained four scenes, and each scene was associated with two fractal objects: a “good” object linked to a liquid reward and a “bad” object yielding no reward. For Scenes 1 and 2, object-reward contingencies were stable, while for Scenes 3 and 4, the values were reversed. This flexible-value design allowed us to dissociate neuronal responses to object value from those driven by low-level visual features.

A typical trial began with a 1000-ms scene presentation, followed by a 700-ms central fixation period. Subsequently, a good or bad object appeared at one of six peripheral locations (15° eccentricity). To accept the object, the monkey had to make a saccade to it and maintain fixation for at least 400 ms, which resulted in a reward (0.4 mL of juice) for a good object. To reject an object, the monkey could use one of three strategies: a brief saccade to the object followed by a return to center (“return”), maintaining central fixation (“stay”), or a saccade to a location away from both the object and the center (“other”).

#### MRI and Localization

Following chamber implantation, MRI scans were performed to map anatomical structures relative to the recording grid. Recording sites were localized using a 3-T MRI system (MAGNETOM Prisma, Siemens) to acquire three-dimensional T1-weighted (T1w) and T2-weighted (T2w) sequences at 0.5-mm isotropic resolution.

To enhance the visualization of the STN, we employed quantitative susceptibility mapping (QSM), which provides superior contrast for iron-rich subcortical structures compared to conventional imaging.^42–44^ QSM images were reconstructed from phase images acquired with a 3D multi-echo gradient echo sequence (repetition time: 50 ms; echo times: 3.7, 10.1, 16.7, 23.4, 30.0, 36.6, 43.2 ms).^41^ The reconstruction pipeline, implemented in MATLAB 2019 using the morphology-enabled dipole inversion toolbox, included phase unwrapping, background field removal, and dipole inversion to generate the final susceptibility maps.^45^

To precisely locate the grid holes, a high-resolution T1w MPRAGE sequence (0.33 × 0.33 × 0.35 mm^3^ voxel size) was acquired while the grid was filled with a gadolinium-based contrast agent. For recording guidance, we created fused images combining this high-resolution scan with the QSM or conventional T1w images. To illustrate the recording locations (Figure S2D), the STN was automatically segmented on each monkey’s T1w image using an AFNI-based pipeline, which aligned the native brain to a standard macaque template (NMT v2.0) and then inversely transformed the Subcortical Atlas of the Rhesus Macaque (SARM).^46–49^ The accuracy of this segmentation was confirmed by comparison with the QSM images, which provide superior visualization of the STN structure compared to standard T1w and T2w images.^44,50^ Final 3D visualizations were created using 3D Slicer (v5.2.2)^51^ and Blender (v3.5) software.

#### Electrophysiology

We began single-unit recordings in the STN after the monkeys achieved stable task performance, defined as >90% accuracy in discriminating between good and bad objects. We used tungsten microelectrodes (1–9 MΩ; FHC; Alpha Omega) that were advanced through stainless steel guide tubes using a hydraulic micromanipulator (Narishige). The recorded neuronal signals were amplified, bandpass filtered (0.3–10 kHz; A-M Systems), and digitized at 40 kHz. Individual units were isolated online using custom voltage-time window discrimination software based on waveform morphology. A neuron was selected for recording if it exhibited clear task-related modulation following target onset and could be well-isolated from background noise and other units. Single neurons were isolated online using a voltage-time window discriminator, where units were separated based on both their waveform amplitude and specific shape using a combination of inclusion and exclusion windows.

### QUANTIFICATION AND STATISTICAL ANALYSIS

All data were preprocessed and analyzed using MATLAB 2022b and RStudio.^52^ The sample size was determined based on previous studies recording from the primate STN.^32^

#### Behavioral Data Analysis

Saccade onsets were detected when eye velocity surpassed 40°/s. Reaction times (Figure S1A) were calculated for initial saccades to objects and were compared between conditions using the Welch t-test. The proportion of “stay” responses between stable- and flexible-value scenes was compared using Fisher’s exact test.

#### Neuronal Data Analysis

Spike trains were aligned to scene, target, and saccade onsets. Peristimulus time histograms (PSTHs) were constructed with 1-ms bins and smoothed with a Gaussian kernel (σ = 20 ms). For population-level analyses, neuronal activity was z-transformed by subtracting the mean firing rate during a 500-ms pre-event baseline period and dividing by the standard deviation of that baseline activity.^53,54^

#### Clustering Analysis

Neurons were classified using k-means clustering based on their average z-scored firing rates in a 200-ms window (100–300 ms) after the presentation of contralateral good and bad objects. To determine the optimal number of clusters (K), we used the silhouette method. We simulated silhouette values 5,000 times for K ranging from 2 to 6 and selected the K that yielded the highest average value (Figure S2A).

#### Principal Component and Clustering Analysis

To validate the functional groupings identified by the window-based method and to characterize the neural population in a more data-driven manner, we performed PCA on the trial-averaged neural activity.

The input matrix for the PCA was constructed from the Z-scored activity on contralateral trials. To treat the responses to different stimulus values independently, the average responses to ‘good’ and ‘bad’ objects for each neuron were stacked as separate entries, resulting in a matrix with 374 rows (187 neurons × 2 conditions). This analysis was performed on a short window of activity (0 to 300 ms post-stimulus). PCA was performed using the *prcomp* function in R, with the data for each time point centered and scaled. A cumulative variance plot was generated to determine the number of components to retain for subsequent analysis, which indicated that the first three PCs collectively captured the majority (>60%) of the structured variance in the data (Figure S3B).

To subsequently group neurons based on the functional properties revealed by these top three PCs, we applied a k-means clustering algorithm. The feature space for clustering was derived from the PCA scores (weights). For each of the 187 neurons, we extracted its scores on the top three principal components (PC1-3) for both the ‘good’ and ‘bad’ conditions. These were combined to form a six-dimensional feature vector for each neuron. K-means clustering (*kmeans* function in R) was then applied to this 187-neuron × 6-feature matrix.

#### Linear Mixed-Effects Models (LMMs)

To compare normalized neuronal activity across different conditions (Figures 3 and 4), we used LMMs to reduce type I errors and better represent the data structure.^55^ These models included fixed effects for the experimental variables and their interactions, with random intercepts for monkey and for neuron nested within monkey to account for non-independence of the data. The general structure of the most complex model, using R’s formula notation, was:

NormalizedActivity~Scene∗Value∗Direction+(1|MonkeyID)+(1|MonkeyID:NeuronID)

Model significance was assessed using a parametric bootstrap method (10,000 iterations) comparing the full model to a null model containing only the random effects. Significant main effects or interactions were followed by post-hoc pairwise t-tests with Bonferroni correction. These analyses were performed in RStudio using the lme4, pbkrtest, and emmeans packages.^56–58^

#### Relationship between Neuronal Activity and Reaction Time

To examine the trial-by-trial relationship between neuronal activity and RT, we focused on ‘accept’ trials for contralateral good objects. For each trial, we calculated the z-scored SDF (Spike Density Functions) and extracted three parameters: 1) Response Onset Latency (the first time point after target onset where the z-score exceeded 2), 2) Peak Response Magnitude (the maximum z-score within a 150-ms window following the response onset), and 3) Response Slope (the rate of change in z-score from onset to peak).

To statistically assess the relationship between each neuronal parameter and RT, we fitted separate LMMs. These models included the neuronal parameter as a fixed effect to predict RT, with random intercepts for MonkeyID and NeuronID to account for non-independence of the data. The general structure of the models, consistent with our R script implementation, was:

RT∼NeuronalParameter+(1|MonkeyID)+(1|MonkeyID:NeuronID)

The significance of the fixed effect was assessed based on the *P*-value derived from Satterthwaite’s method for approximating degrees of freedom. A Bonferroni correction was applied to account for the nine separate models fitted, resulting in a significance threshold of *P* < 0.0056. These analyses were performed in RStudio using the *lme4* and *lmerTest* packages. For visualization in Figure 4G, each scatter plot was overlaid with the predicted marginal effect line and its 95% confidence interval derived from the LMM. The unstandardized regression coefficient (β) and its *P*-value from the model were displayed on each plot.

#### Sliding-window General Linear Model (GLM)

To quantify how the choice signal of individual neurons evolved over time, we performed a trial-by-trial analysis using a sliding-window GLM on contralateral trials.^59^ For each neuron, a 50-ms analysis window was moved in 1-ms steps across the trial epoch. At each step, we fitted a linear model (*lm* function in R) of the form:

$$\text{MeanActivity}=\beta_{0}+\beta_{\text{Action}}*\text{Action}+\epsilon$$

where Action was coded as 1 for ‘accept’ and −1 for ‘reject’. The β coefficient for the Action term was extracted at each time point. For visualization (Figure 5A), these β coefficients were smoothed (21-ms moving average) and sorted by response preference and latency.

#### Population-level Analysis using PCA

To visualize population dynamics, we used PCA for dimensionality reduction. For each neuron, spike density functions (SDFs; σ = 20 ms) from contralateral trials were baseline-corrected. Trial-averaged SDFs for ‘accept’ and ‘reject’ conditions were combined to fit a single PCA model. The population activity for each condition was then projected onto the first three PCs, which captured the largest amount of variance in the data. To statistically determine the time of choice signal emergence, we calculated the Euclidean distance between the two trajectories in PC space at each time step. The divergence time was defined as the first time point post-target where this distance exceeded three standard deviations above the mean baseline distance.

#### Population Decoding using Elastic Net

To quantify the predictive power of the STN population, we used an Elastic Net regularized regression approach. For each trial, spike counts in 1-ms bins (−600 to 600 ms from target onset) across all neurons were concatenated into a feature vector. ^60^ All feature standardization (z-scoring) was fit exclusively on the training data within each cross-validation fold and then applied to the corresponding test sets to prevent information leakage.

To obtain an unbiased estimate of model performance and prevent data leakage from non-independent trials, we assessed performance using a nested, 10-fold cross-validation procedure, grouped by neuron. In the outer loop, the dataset was split into 10 folds such that all trials from a given neuron belonged to the same fold. For each iteration of the outer loop, 9 folds were used for training, and the remaining fold was held out for testing. Within the training set, an inner 10-fold cross-validation (also grouped by neuron) was performed to select the optimal regularization parameter (*λ*) that maximized the area under the ROC curve (*AUC*) for choice prediction or minimized mean-squared error (MSE) for RT prediction. A final model was then trained on the entire outer training set using this optimal λ and evaluated on the held-out test fold. The overall performance metric (*AUC* or *R^2^*) was calculated from the aggregated predictions from all 10 outer test folds. We set the Elastic Net mixing parameter α to 0.5 a priori, while λ was selected in the inner cross-validation loop.

To assess the importance of each cluster, we performed an ablation analysis (systematically removing one cluster at a time) and tested the performance of each cluster individually, applying the same nested, grouped cross-validation procedure to each subset of data. The statistical significance of the single-cluster *AUCs* was determined via a permutation test (2,000 iterations). In each iteration, choice labels were shuffled, and the entire nested, grouped cross-validation procedure was repeated to generate an accurate null distribution of *AUCs*. This entire set of decoding analyses was conducted first on the data pooled from both monkeys and then repeated on the data from each monkey individually to confirm the consistency of the findings.

## Supplementary Material

1

## Data Availability

This study did not generate new unique reagents.

## Data and Code Availability

Any additional information required to reanalyze the data reported in this paper is available from the lead contact upon request.
