# Supplementary material for: Beyond the Brake: the Subthalamic Nucleus Predominantly Facilitates Action in Non-human Primates
Source: bioRxiv. 2025 Dec 12:2025.12.09.692702. Preprint. [Version 1] doi: 10.64898/2025.12.09.692702 (PMC12713583; doi:10.64898/2025.12.09.692702)
Supplement: 1 [file NIHPP2025.12.09.692702v1-supplement-1.pdf]

bioRxiv preprint doi: https://doi.org/10.64898/2025.12.09.692702; this version posted December 12, 2025. The copyright holder for this preprint (which was not certified by peer review) is the author/funder. This article is a US Government work. It is not subject to copyright under 17 USC 105 and is also made available for use under a CC0 license.

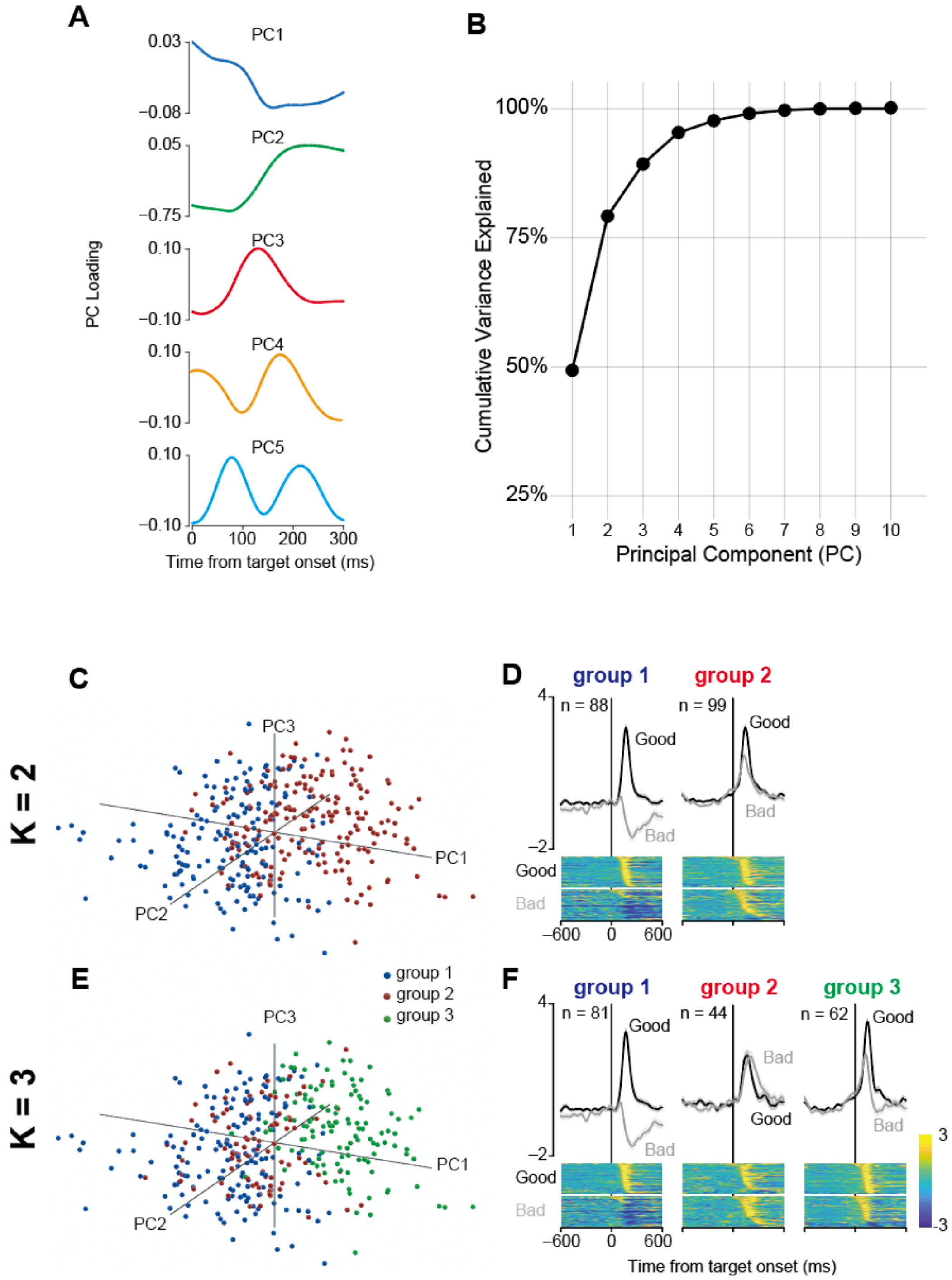

Figure S3. PCA-based functional grouping of STN neurons using post-stimulus activity.

(A) Temporal waveforms of the top five principal components (PCs) derived from the neural activity in the 0–300 ms post-stimulus window. The x-axis represents time from target onset, and the y-axis represents the PC loading.

bioRxiv preprint doi: https://doi.org/10.64898/2025.12.09.692702; this version posted December 12, 2025. The copyright holder for this preprint (which was not certified by peer review) is the author/funder. This article is a US Government work. It is not subject to copyright under 17 USC 105 and is also made available for use under a CC0 license.

(B) Cumulative variance plot. The curve shows the cumulative percentage of total variance explained as a function of the number of principal components included.
(C) 3D scatter plot of the neural population in the space defined by the top three PCs. Each point represents a single neuron-condition, colored by its cluster assignment for the K=2 solution.
(D) Population activity for the two functional groups identified at K=2. Top panels show the mean normalized activity (± SEM) for contralateral good (black) and bad (gray) trials. Bottom panels show heatmaps of the Z-scored activity of all individual neurons within each group, sorted by response latency.
(E) Same as (C), but for the K=3 clustering solution.
(F) Same as (D), but for the three functional groups identified at K=3.

bioRxiv preprint doi: https://doi.org/10.64898/2025.12.09.692702; this version posted December 12, 2025. The copyright holder for this preprint (which was not certified by peer review) is the author/funder. This article is a US Government work. It is not subject to copyright under 17 USC 105 and is also made available for use under a CC0 license.

Supplementary information

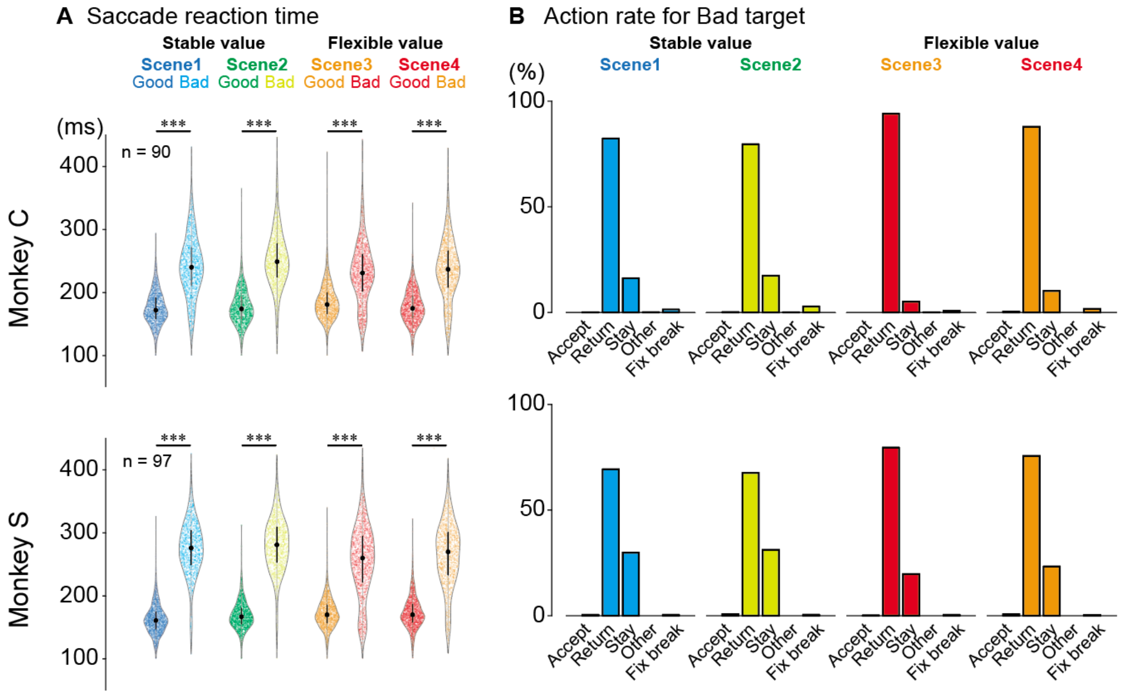

Figure S1. Behavioral performance.

(A) Saccadic reaction times for good and bad objects in the two monkeys (C and S). Violin plots show that across all scenes and for both monkeys, saccades to good objects had significantly shorter latencies than saccades to bad objects during the return response (****P* < 0.0001 for all comparisons, the Welch t-tests). Each dot represents data from a single recorded neuron (n = 90 for monkey C and n = 97 for monkey S). The thick horizontal line indicates the median, the box indicates the interquartile range (IQR), and the whiskers extend to the most extreme data point within 1.5 times the IQR. See also Table S1.

(B) Proportions of different behavioral responses to bad objects for each scene. Both monkeys exhibited similar patterns: incorrect acceptance of bad objects was rare, and rejection responses consisted predominantly of return saccades, followed by stay and other responses. Accept incorrect acceptance of bad objects; Return, return saccades; Stay, maintaining central fixation; Other, saccades away from both the object and the central fixation point; Fix break, fixation break error. See also Table S2.

Abbreviations: FP, fixation point; ITI, inter-trial interval; Obj, object; Tgt, target.

bioRxiv preprint doi: https://doi.org/10.64898/2025.12.09.692702; this version posted December 12, 2025. The copyright holder for this preprint (which was not certified by peer review) is the author/funder. This article is a US Government work. It is not subject to copyright under 17 USC 105 and is also made available for use under a CC0 license.

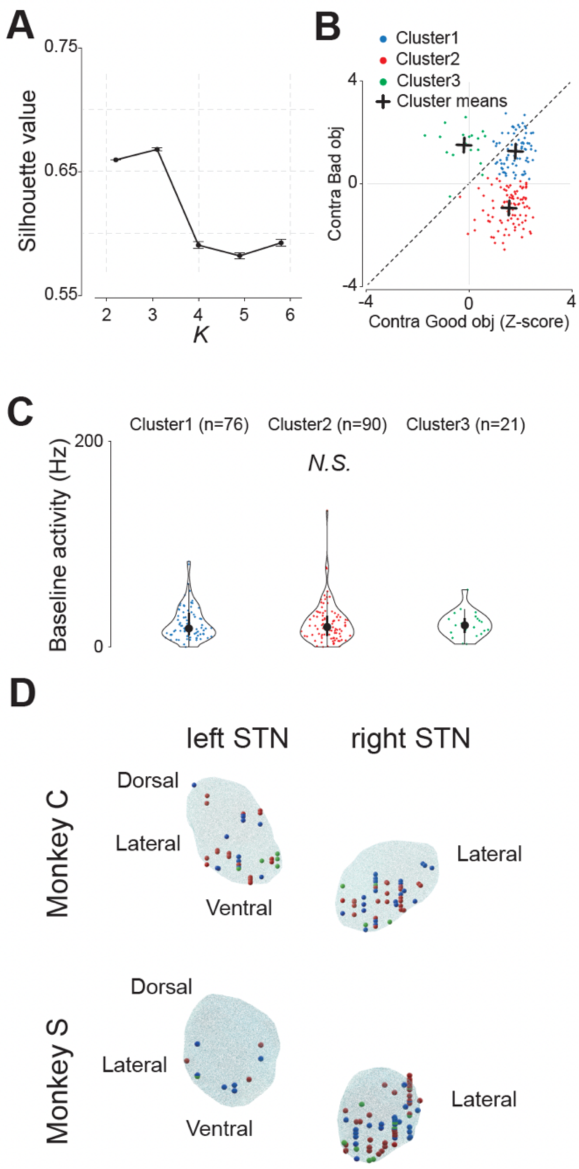

Figure S2. Classification and anatomical location of STN neuron clusters. (A) Optimal cluster number (K) determination. The plot shows the mean silhouette value (±SD) across 5,000 simulations for different values of K. The analysis indicated that K=3 was optimal for partitioning the neuronal population. (B) Functional classification of STN neurons. Each point represents a single neuron, plotted according to its average z-scored firing rate in response to contralateral good (x-axis) and bad (y-axis) objects (100-300 ms post-object onset). Colors denote cluster assignment (Cluster 1: blue; Cluster 2: red; Cluster 3: green), and crosses indicate the cluster means.

bioRxiv preprint doi: https://doi.org/10.64898/2025.12.09.692702; this version posted December 12, 2025. The copyright holder for this preprint (which was not certified by peer review) is the author/funder. This article is a US Government work. It is not subject to copyright under 17 USC 105 and is also made available for use under a CC0 license.

(C) Baseline firing rates for each cluster. Violin plots show the distribution of baseline activity (500-ms period before scene onset). No significant difference was found among the clusters (Kruskal–Wallis test, $P > 0.05$). Plot conventions: circle, median; thick line, interquartile range (IQR); thin line, 1.5 × IQR.
(D) Anatomical distribution of recorded neurons. 3D reconstructions of the STN show the recording locations for each neuron in Monkey C (top) and Monkey S (bottom), color-coded by cluster identity. These 3D maps are viewed from the top-left.

Table S1. Saccade reaction times in each condition of each monkey.

**Monkey Cr**	n	mean RT (ms)	SD	95% CI
**Good obj**				
ObjA in Scene1	1912	175.7	26.5	[174.5 176.9]
ObjC in Scene2	1900	176.8	27.5	[175.5 178.0]
ObjE in Scene3	1931	184.9	27.9	[183.7 186.2]
ObjF in Scene4	1893	177.7	25.9	[176.6 178.9]
**Bad obj**				
ObjB in Scene1	1719	241.8	49.2	[239.4 244.1]
ObjD in Scene2	1539	252.1	46.1	[249.8 254.4]
ObjF in Scene3	1924	229.9	49.1	[227.7 232.1]
ObjE in Scene4	1671	235.8	49.3	[233.4 238.2]
**Monkey Sp**	n	mean RT (ms)	SD	95% CI
**Good obj**				
ObjA in Scene1	1991	163.0	24.0	[162.0 164.1]
ObjC in Scene2	1981	169.5	23.3	[168.5 170.6]
ObjE in Scene3	1974	172.5	24.3	[171.4 173.6]
ObjF in Scene4	1958	173.2	25.2	[172.1 174.3]
**Bad obj**				
ObjB in Scene1	1466	274.6	44.9	[272.3 276.9]
ObjD in Scene2	1460	280.2	44.8	[277.9 282.5]
ObjF in Scene3	1713	255.5	57.7	[252.8 258.3]
ObjE in Scene4	1522	264.6	52.9	[261.9 267.3]

Table S2. Counts of chosen actions for Bad objects

**Monkey Ch**	total	accept	Return	Stay	other	fxbreak
Scene1	545	0	412	92	17	24
Scene2	502	0	424	51	9	18
Scene3	469	0	410	41	1	17
Scene4	547	0	462	63	7	15
non-switch(scene1,2)	1044	0	836	143	26	42
switch(scene3,4)	1016	0	872	104	8	32

**Monkey Cr**	total	accept	Return	Stay	other	fxbreak
Scene1	1050	1	872	156	1	20
Scene2	996	2	802	159	1	32
Scene3	978	15	903	50	1	9
Scene4	1051	14	902	110	2	23
non-switch(scene1,2)	2046	3	1674	314	2	52
switch(scene3,4)	2029	29	1805	160	3	32

**Monkey Sp**	total	accept	Return	Stay	other	fxbreak
Scene1	866	2	581	278	0	5
Scene2	842	1	557	282	0	2
Scene3	923	4	692	225	0	2
Scene4	946	2	729	213	0	2
non-switch(scene1,2)	1708	3	1138	560	0	7
switch(scene3,4)	1869	6	1421	438	0	4

Table S3. Summary of statistical test to compare the normalized neuronal activity of STN neurons of cluster1 at target onset among conditions during choice task in Figure 3.

Cluster1						
parametric bootstrap test (n = 10,000)	*p*					
full model vs. null model	< .001					
post hoc (pairwise t–test, Bonferroni correction)	Mean (SD)	Mean (SD)	*t*	*p*	95% CI	effect size
**Scene1**						
(good, contra) vs (bad, contra)	1.85(0.52)	1.25(0.76)	5.39	<.0001	[0.38, 0.82]	0.87
(good, contra) vs (good, ipsi)	1.85(0.52)	1.09(1.05)	6.82	<.0001	[0.54, 0.98]	1.11
(good, contra) vs (bad, ipsi)	1.85(0.52)	0.91(1.06)	8.45	<.0001	[0.72, 1.16]	1.37
(bad, contra) vs (good, ipsi)	1.25(0.76)	1.09(1.05)	1.43	= 1.55 x 10^–1	[–0.06, 0.38]	0.23
(bad, contra) vs (bad, ipsi)	1.25(0.76)	0.91(1.06)	3.06	= 2.30 x 10^–3	[0.12, 0.56]	0.50
(good, ipsi) vs (bad, ipsi)	1.09(1.05)	0.91(1.06)	1.63	= 1.03 x 10^–1	[–0.04, 0.40]	0.27
**Scene2**						
(good, contra) vs (bad, contra)	1.86(0.57)	1.09(0.87)	6.87	<.0001	[0.55, 0.99]	1.12
(good, contra) vs (good, ipsi)	1.86(0.57)	1.14(0.93)	6.47	<.0001	[0.50, 0.94]	1.05
(good, contra) vs (bad, ipsi)	1.86(0.57)	0.78(1.16)	9.67	<.0001	[0.86, 1.30]	1.58
(bad, contra) vs (good, ipsi)	1.09(0.87)	1.14(0.93)	–0.42	= 6.75 x 10^–1	[–0.27, 0.17]	–0.07
(bad, contra) vs (bad, ipsi)	1.09(0.87)	0.78(1.16)	2.82	= 4.90 x 10^–3	[0.10, 0.54]	0.46
(good, ipsi) vs (bad, ipsi)	1.14(0.93)	0.78(1.16)	3.25	= 1.20 x 10^–3	[0.14, 0.58]	0.53
**Scene3**						
(good, contra) vs (bad, contra)	1.80(0.57)	1.29(0.84)	4.65	<.0001	[0.30, 0.74]	0.75
(good, contra) vs (good, ipsi)	1.80(0.57)	1.11(1.06)	6.25	<.0001	[0.48, 0.91]	1.01
(good, contra) vs (bad, ipsi)	1.80(0.57)	0.97(1.07)	7.52	<.0001	[0.62, 1.06]	1.22
(bad, contra) vs (good, ipsi)	1.29(0.84)	1.11(1.06)	1.61	= 1.08 x 10^–1	[–0.04, 0.40]	0.26
(bad, contra) vs (bad, ipsi)	1.29(0.84)	0.97(1.07)	2.87	= 4.20 x 10^–3	[0.10, 0.54]	0.47
(good, ipsi) vs (bad, ipsi)	1.11(1.06)	0.97(1.07)	1.26	= 2.07 x 10^–1	[–0.08, 0.36]	0.20
**Scene4**						
(good, contra) vs (bad, contra)	1.87(0.50)	1.28(0.79)	5.25	<.0001	[0.37, 0.80]	0.85
(good, contra) vs (good, ipsi)	1.87(0.50)	1.14(1.06)	6.53	<.0001	[0.51, 0.95]	1.06
(good, contra) vs (bad, ipsi)	1.87(0.50)	1.00(1.07)	7.76	<.0001	[0.65, 1.09]	1.26
(bad, contra) vs (good, ipsi)	1.28(0.79)	1.14(1.06)	1.28	= 2.02 x 10^–1	[–0.08, 0.36]	0.21
(bad, contra) vs (bad, ipsi)	1.28(0.79)	1.00(1.07)	2.53	= 1.17 x 10^–2	[0.06, 0.50]	0.41
(good, ipsi) vs (bad, ipsi)	1.14(1.06)	1.00(1.07)	1.25	= 2.10 x 10^–1	[–0.08, 0.36]	0.20

Table S4. Summary of statistical test to compare the normalized neuronal activity of STN neurons of cluster2 at target onset among conditions during choice task in Figure 3.

Cluster2						
parametric bootstrap test (n = 10,000)	*p*					
full model vs. null model	< .001					
post hoc (pairwise t–test, Bonferroni correction)	Mean (SD)	Mean (SD)	*t*	*p*	95% CI	effect size
**Scene1**						
(good, contra) vs (bad, contra)	1.55(0.64)	–1.21(0.94)	25.72	<.0001	[2.55, 2.97]	3.83
(good, contra) vs (good, ipsi)	1.55(0.64)	0.64(1.05)	8.47	<.0001	[0.70, 1.12]	1.26
(good, contra) vs (bad, ipsi)	1.55(0.64)	–1.15(0.95)	25.18	<.0001	[2.49, 2.92]	3.75
(bad, contra) vs (good, ipsi)	–1.21(0.94)	0.64(1.05)	–17.25	<.0001	[–2.06, –1.64]	–2.57
(bad, contra) vs (bad, ipsi)	–1.21(0.94)	–1.15(0.95)	–0.54	= 5.89 x 10^–1	[–0.27, 0.15]	–0.08
(good, ipsi) vs (bad, ipsi)	0.64(1.05)	–1.15(0.95)	16.71	<.0001	[1.58, 2.01]	2.49
**Scene2**						
(good, contra) vs (bad, contra)	1.60(0.66)	–1.09(0.82)	25.06	<.0001	[2.48, 2.90]	3.74
(good, contra) vs (good, ipsi)	1.60(0.66)	0.71(0.98)	8.35	<.0001	[0.69, 1.11]	1.25
(good, contra) vs (bad, ipsi)	1.60(0.66)	–1.12(0.85)	25.35	<.0001	[2.51, 2.93]	3.78
(bad, contra) vs (good, ipsi)	–1.09(0.82)	0.71(0.98)	–16.70	<.0001	[–2.00, –1.58]	–2.49
(bad, contra) vs (bad, ipsi)	–1.09(0.82)	–1.12(0.85)	0.29	= 7.70 x 10^–1	[–0.18, 0.24]	0.04
(good, ipsi) vs (bad, ipsi)	0.71(0.98)	–1.12(0.85)	17.00	<.0001	[1.61, 2.04]	2.53
**Scene3**						
(good, contra) vs (bad, contra)	1.68(0.59)	–0.90(0.91)	23.94	<.0001	[2.37, 2.79]	3.58
(good, contra) vs (good, ipsi)	1.68(0.59)	0.77(0.98)	8.49	<.0001	[0.70, 1.12]	1.27
(good, contra) vs (bad, ipsi)	1.68(0.59)	–1.16(0.90)	26.38	<.0001	[2.63, 3.05]	3.94
(bad, contra) vs (good, ipsi)	–0.90(0.91)	0.77(0.98)	–15.48	<.0001	[–1.88, –1.46]	–2.31
(bad, contra) vs (bad, ipsi)	–0.90(0.91)	–1.16(0.90)	2.43	= 1.51 x 10^–2	[0.05, 0.48]	0.36
(good, ipsi) vs (bad, ipsi)	0.77(0.98)	–1.16(0.90)	17.92	<.0001	[1.72, 2.14]	2.68
**Scene4**						
(good, contra) vs (bad, contra)	1.64(0.59)	–0.93(0.93)	23.82	<.0001	[2.35, 2.78]	3.56
(good, contra) vs (good, ipsi)	1.64(0.59)	0.64(0.98)	9.33	<.0001	[0.79, 1.21]	1.39
(good, contra) vs (bad, ipsi)	1.64(0.59)	–1.13(0.82)	25.76	<.0001	[2.56, 2.98]	3.84
(bad, contra) vs (good, ipsi)	–0.93(0.93)	0.64(0.98)	–14.51	<.0001	[–1.77, –1.35]	–2.17
(bad, contra) vs (bad, ipsi)	–0.93(0.93)	–1.13(0.82)	1.87	= 6.18 x 10^–2	[–0.01, 0.41]	0.28
(good, ipsi) vs (bad, ipsi)	0.64(0.98)	–1.13(0.82)	16.43	<.0001	[1.55, 1.98]	2.45

Table S5. Summary of statistical test to compare the normalized neuronal activity of STN neurons of cluster3 at target onset among conditions during choice task in Figure 3.

Cluster3						
parametric bootstrap test (n = 10,000)	*p*					
full model vs. null model	< .001					
post hoc (pairwise t–test, Bonferroni correction)	Mean (SD)	Mean (SD)	*t*	*p*	95% CI	effect size
**Scene1**						
(good, contra) vs (bad, contra)	0.19(0.81)	1.49(0.86)	–5.60	<.0001	[–1.76, –0.84]	–1.73
(good, contra) vs (good, ipsi)	0.19(0.81)	–0.25(0.84)	1.87	= 6.29 x 10^–2	[–0.02, 0.89]	0.58
(good, contra) vs (bad, ipsi)	0.19(0.81)	1.17(0.87)	–4.25	<.0001	[–1.44, –0.53]	–1.31
(bad, contra) vs (good, ipsi)	1.49(0.86)	–0.25(0.84)	7.46	<.0001	[1.28, 2.19]	2.30
(bad, contra) vs (bad, ipsi)	1.49(0.86)	1.17(0.87)	1.35	= 1.78 x 10^–1	[–0.14, 0.77]	0.42
(good, ipsi) vs (bad, ipsi)	–0.25(0.84)	1.17(0.87)	–6.11	<.0001	[–1.88, –0.96]	–1.89
**Scene2**						
(good, contra) vs (bad, contra)	–0.17(0.82)	1.38(1.01)	–6.67	<.0001	[–2.01, –1.09]	–2.06
(good, contra) vs (good, ipsi)	–0.17(0.82)	–0.18(0.99)	0.06	= 9.55 x 10^–1	[–0.44, 0.47]	0.02
(good, contra) vs (bad, ipsi)	–0.17(0.82)	1.13(0.92)	–5.57	<.0001	[–1.75, –0.84]	–1.72
(bad, contra) vs (good, ipsi)	1.38(1.01)	–0.18(0.99)	6.73	<.0001	[1.11, 2.02]	2.08
(bad, contra) vs (bad, ipsi)	1.38(1.01)	1.13(0.92)	1.10	= 2.74 x 10^–1	[–0.20, 0.71]	0.34
(good, ipsi) vs (bad, ipsi)	–0.18(0.99)	1.13(0.92)	–5.63	<.0001	[–1.77, –0.85]	–1.74
**Scene3**						
(good, contra) vs (bad, contra)	–0.09(0.94)	1.49(1.01)	–6.80	<.0001	[–2.04, –1.12]	–2.10
(good, contra) vs (good, ipsi)	–0.09(0.94)	–0.18(0.72)	0.41	= 6.86 x 10^–1	[–0.36, 0.55]	0.13
(good, contra) vs (bad, ipsi)	–0.09(0.94)	1.15(0.91)	–5.36	<.0001	[–1.70, –0.79]	–1.65
(bad, contra) vs (good, ipsi)	1.49(1.01)	–0.18(0.72)	7.20	<.0001	[1.22, 2.13]	2.22
(bad, contra) vs (bad, ipsi)	1.49(1.01)	1.15(0.91)	1.44	= 1.50 x 10^–1	[–0.12, 0.79]	0.45
(good, ipsi) vs (bad, ipsi)	–0.18(0.72)	1.15(0.91)	–5.76	<.0001	[–1.80, –0.88]	–1.78
**Scene4**						
(good, contra) vs (bad, contra)	0.06(0.67)	1.44(0.94)	–5.92	<.0001	[–1.83, –0.92]	–1.83
(good, contra) vs (good, ipsi)	0.06(0.67)	–0.19(0.85)	1.08	= 2.80 x 10^–1	[–0.21, 0.71]	0.33
(good, contra) vs (bad, ipsi)	0.06(0.67)	1.03(1.03)	–4.17	<.0001	[–1.43, –0.51]	–1.29
(bad, contra) vs (good, ipsi)	1.44(0.94)	–0.19(0.85)	7.00	<.0001	[1.17, 2.09]	2.16
(bad, contra) vs (bad, ipsi)	1.44(0.94)	1.03(1.03)	1.75	= 8.16 x 10^–2	[–0.05, 0.86]	0.54
(good, ipsi) vs (bad, ipsi)	–0.19(0.85)	1.03(1.03)	–5.26	<.0001	[–1.68, –0.77]	–1.62

Table S6. Summary of statistical test to compare the normalized neuronal activity of STN neurons of cluster1 at saccade onset among conditions during choice task in Figure 4.

Cluster1						
parametric bootstrap test (n = 10,000)	*p*					
full model vs. null model	< .001					
post hoc (pairwise t–test, Bonferroni correction)	Mean (SD)	Mean (SD)	*t*	*p*	95% CI	effect size
**Scene1**						
(good, contra) vs (bad, contra)	1.69(0.49)	1.15(0.79)	4.90	<.0001	[0.33, 0.76]	0.79
(good, contra) vs (good, ipsi)	1.69(0.49)	1.13(0.81)	5.03	<.0001	[0.34, 0.78]	0.82
(good, contra) vs (bad, ipsi)	1.69(0.49)	0.77(1.06)	8.33	<.0001	[0.71, 1.15]	1.35
(bad, contra) vs (good, ipsi)	1.15(0.79)	1.13(0.81)	0.13	= 8.95 x 10^–1	[–0.20, 0.23]	0.02
(bad, contra) vs (bad, ipsi)	1.15(0.79)	0.77(1.06)	3.43	= 6.00 x 10^–4	[0.16, 0.60]	0.56
(good, ipsi) vs (bad, ipsi)	1.13(0.81)	0.77(1.06)	3.30	= 1.00 x 10^–3	[0.15, 0.59]	0.54

Table S7. Summary of statistical test to compare the normalized neuronal activity of STN neurons of cluster2 at saccade onset among conditions during choice task in Figure 4.

Cluster2						
parametric bootstrap test (n = 10,000)	*p*					
full model vs. null model	< .001					
post hoc (pairwise t–test, Bonferroni correction)	Mean (SD)	Mean (SD)	*t*	*p*	95% CI	effect size
**Scene1**						
(good, contra) vs (bad, contra)	1.34(0.62)	–1.27(1.03)	24.43	<.0001	[2.41, 2.83]	3.64
(good, contra) vs (good, ipsi)	1.34(0.62)	0.57(0.90)	7.19	<.0001	[0.56, 0.98]	1.07
(good, contra) vs (bad, ipsi)	1.34(0.62)	–1.22(0.93)	23.89	<.0001	[2.35, 2.77]	3.56
(bad, contra) vs (good, ipsi)	–1.27(1.03)	0.57(0.90)	–17.24	<.0001	[–2.06, –1.64]	–2.57
(bad, contra) vs (bad, ipsi)	–1.27(1.03)	–1.22(0.93)	–0.54	= 5.93 x 10^–1	[–0.27, 0.15]	–0.08
(good, ipsi) vs (bad, ipsi)	0.57(0.90)	–1.22(0.93)	16.70	<.0001	[1.58, 2.00]	2.49

Table S8. Summary of statistical test to compare the normalized neuronal activity of STN neurons of cluster3 at saccade onset among conditions during choice task in Figure 4.

Cluster3						
parametric bootstrap test (n = 10,000)	*p*					
full model vs. null model	< .001					
post hoc (pairwise t–test, Bonferroni correction)	Mean (SD)	Mean (SD)	*t*	*p*	95% CI	effect size
**Scene1**						
(good, contra) vs (bad, contra)	0.62(0.88)	1.36(0.82)	–3.27	= 1.20 x 10^–3	[–1.19, –0.30]	–1.01
(good, contra) vs (good, ipsi)	0.62(0.88)	0.21(0.99)	1.81	= 7.19 x 10^–2	[–0.04, 0.86]	0.56
(good, contra) vs (bad, ipsi)	0.62(0.88)	1.14(0.98)	–2.29	= 2.28 x 10^–2	[–0.97, –0.07]	–0.71
(bad, contra) vs (good, ipsi)	1.36(0.82)	0.21(0.99)	5.07	<.0001	[0.71, 1.60]	1.57
(bad, contra) vs (bad, ipsi)	1.36(0.82)	1.14(0.98)	0.98	= 3.29 x 10^–1	[–0.23, 0.67]	0.30
(good, ipsi) vs (bad, ipsi)	0.21(0.99)	1.14(0.98)	–4.10	= 1.00 x 10^–4	[–1.38, –0.48]	–1.26

Table S9. Fixed effects from LMMs models predicting reaction time. Linear mixed–effects models were fitted for each cluster to assess the relationship between neuronal response parameters and reaction time (RT). Models included NeuronID and MonkeyID as random intercepts.

Cluster	Predictor (Fixed Effect)	Estimate (β)	S.E.	95% CI	*t*	*P*
Cluster 1	Response Onset (ms)	0.036	0.01	[0.0160, 0.0552]	3.56	0.0004
	Response Peak (Z–score)	0.006	0.003	[–0.0010, 0.0124]	1.68	0.0931
	Response Slope (Z/ms)	1.037	0.488	[0.0802, 1.9940]	2.12	0.0338
Cluster 2	Response Onset (ms)	0.057	0.009	[0.0383, 0.0749]	6.07	< .0001
	Response Peak (Z–score)	–0.059	0.028	[–0.1133, –0.0056]	–2.16	0.0306
	Response Slope (Z/ms)	–4.04	2.012	[–7.9829, –0.0966]	–2.01	0.0447
Cluster 3	Response Onset (ms)	–0.013	0.023	[–0.0588, 0.0332]	–0.55	0.5856
	Response Peak (Z–score)	–0.572	0.425	[–1.4044, 0.2604]	–1.35	0.1788
	Response Slope (Z/ms)	–32.128	19.425	[–70.2011, 5.9455]	–1.65	0.099
